# Differences in perceptual assimilation following training

**DOI:** 10.1121/10.0003863

**Published:** 2021-04

**Authors:** Heather Kabakoff, Julia Kharlamenko, Erika S. Levy, Susannah V. Levi

**Affiliations:** 1 Department of Communicative Sciences and Disorders, New York University, New York, New York 10012, USA; 2 Department of Biobehavioral Sciences, Teachers College, Columbia University, New York, New York 10027, USA heather.kabakoff@nyu.edu, jk5096@nyu.edu, elevy@exchange.tc.columbia.edu, svlevi@nyu.edu

## Abstract

Learning to perceive non-native speech sounds is difficult for adults. One method to improve perception of non-native contrasts is through a distributional learning paradigm. Three groups of native-English listeners completed a perceptual assimilation task in which they mapped French vowels onto English vowel categories: Two groups (bimodal, unimodal distribution) completed a perceptual learning task for the French /œ/-/o/ contrast and a third completed no training. Both trained groups differed from the untrained group, but participants in the bimodal group showed a different perceptual mapping for the targeted /œ/ vowel, suggesting that the bimodal condition may maximize perception of non-native contrasts.

## 1. Introduction

Attaining native-like proficiency in a second language is difficult for most adults. Decades of research indicate that young age is a critical factor in second language learning (Baker *et al.*, 2002; Bever, 1981; Flege *et al.*1999). Once the phonetic categories of one's native language are solidified, the ability to perceptually distinguish non-native sounds decreases (Flege, 1995; Iverson *et al.*, 2003; Kuhl, 2000; Michaels, 1974). In addition, the perception of non-native speech sounds is influenced by similarities between the speech sounds in the first and second languages and by the degree and type of exposure to and familiarity with the second language (Levy, 2009a; Levy and Strange, 2008). In the current study, we examined listeners' perceptual organization of non-native vowels following a brief perceptual learning paradigm (distributional learning). The organization patterns following this learning paradigm were assessed through a perceptual assimilation task (Best, 1995) in which listeners mapped speech sounds of a non-native language onto labels in their first language.

### 1.1 Distributional learning

**Fig. 1.**
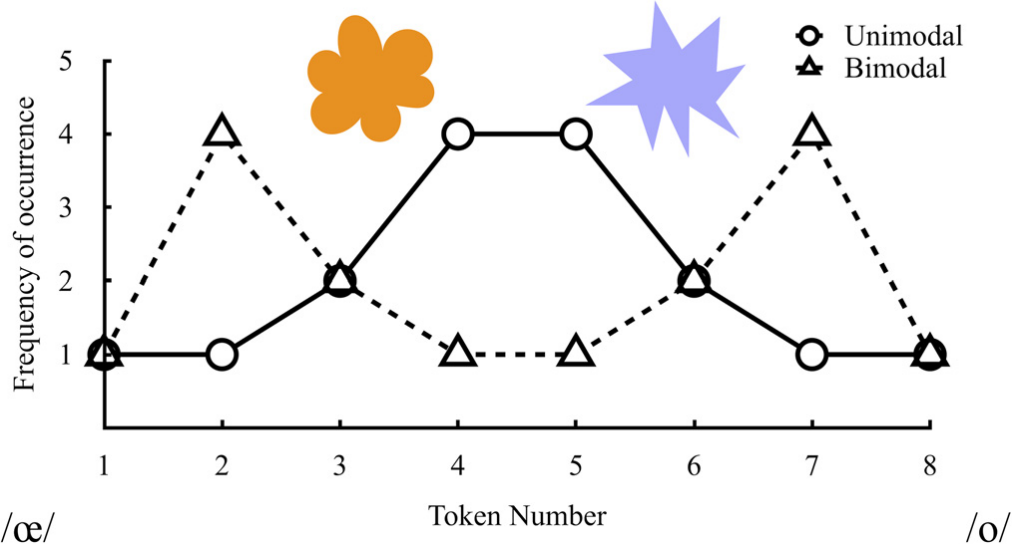
Eight-step acoustic continuum (*x* axis) and frequency of administration of each step (*y* axis) for the unimodal and bimodal training conditions. The depicted mapping is for steps 1–4 to correspond with the orange cloud and steps 5–8 to correspond with the lavender spike shape. Reprinted from Kabakoff *et al.* (2020) with permission.

Researchers have examined ways to improve non-native speech sound perception through various perceptual learning paradigms. One paradigm that has been used with both infants and adults is a distributional learning paradigm that harnesses listeners' ability to track statistical information in the input distribution (Baese-Berk, 2010, 2019; Escudero and Williams, 2014; Hayes, 2003; Kabakoff *et al.*, 2020; Maye and Gerken, 2000; Maye *et al.*, 2002). In these studies, listeners were presented with stimuli along an acoustic continuum, such as the eight-step continuum in Fig. 1. Listeners in a bimodal distribution condition hear more tokens near the endpoints of the continuum (e.g., steps 2 and 7), whereas listeners in a unimodal condition hear more tokens near the center of the continuum (e.g., steps 4 and 5).

Most of these studies have used passive listening exposure, wherein listeners hear the speech sounds and simply check a box marking that a stimulus was played (Maye and Gerken, 2000) or press a key to play the next stimulus (Baese-Berk, 2010). Collectively, the majority of studies have found that hearing many repetitions of stimuli near the endpoints of the continuum (bimodal distribution) facilitates the formation of two novel speech sound categories, whereas hearing stimuli from the center of the distribution (unimodal distribution) does not lead to learning of the two-category structure or leads to less learning of two categories than in the bimodal condition (Baese-Berk, 2010; Hayes, 2003; Maye and Gerken, 2000; Maye *et al.*, 2002). In a recent study, Kabakoff *et al.* (2020) adjusted these passive learning paradigm reported in previous studies by providing adult listeners with feedback during the perceptual learning phase. When provided with feedback, listeners in the unimodal group were also able to learn a novel speech sound contrast, although the learning occurred more gradually for those in the unimodal condition. As described in Kabakoff *et al.* (2020), past studies incorporating passive distributional learning paradigms to represent how new speech sound categories are formed have evolved into a line of research attempting to optimize training paradigms for learning non-native contrasts. As such, the plentitude of studies demonstrating the effectiveness of feedback when learning new speech sound categories supports the addition of feedback to previous distributional training approaches (Goudbeek *et al.*, 2008; Harmon *et al.*, 2019; McCandliss *et al.*, 2002).

### 1.2 Perceptual assimilation

According to the perceptual assimilation model (PAM) (Best, 1995) and PAM-L2 (Best and Tyler, 2007), adults perceive the speech sounds of a non-native language based on similarities and differences with their native language. Perceptual assimilation tasks reveal how non-native speech sounds are mapped onto native sound categories. Previous studies using this task have found that several factors affect this mapping including language experience (Escudero and Boersma, 2002; Levy, 2009a), phonetic environment of the stimulus (Levy, 2009a), prosodic context of the stimulus [i.e., presented in isolation, syllable, or sentence (Strange *et al.*, 2004)], and tenseness of the vowel stimulus (Polka, 1995).

One factor relevant to the current study is listeners' language experience. Escudero and Boersma (2002) found that Dutch listeners with minimal Spanish experience assimilated the Spanish vowels /e/ and /i/ into three Dutch categories /ɛ/-/ɪ/-/i/, whereas intermediate, advanced, and bilingual groups only mapped these vowels onto the two Dutch vowels /ɛ/ and /i/. Similarly, Levy (2009a) found that the French open-mid front rounded vowel /œ/ was assimilated to a variety of American English (AE) vowels (/u/, /ʊ/, /o/, /ʌ/, /ɝ/) by less experienced listeners, and to a smaller set of vowels (approximately 65% of stimuli mapped onto /ʊ/) by more experienced listeners. Levy (2009a) argued that differences in phonetic representation exist among groups with varying levels of language experience, and that these differences affect how listeners map French vowels onto AE vowels. In both of these studies, extensive language experience resulted in more localized perceptual assimilation responses, such that a non-native speech sound corresponded more consistently to one native speech sound category.

### 1.3 The current study

Based on previous distributional training studies that tested perceptual identification and discrimination, we were interested in whether perceptual assimilation patterns would also be altered following distributional training. Namely, we were interested in whether listeners who completed short-term perceptual training would demonstrate different assimilation patterns from untrained listeners. Given previous studies showing differences in perceptual learning between bimodal and unimodal training conditions, we also were interested in whether the two training groups would differ from each other. This direction of inquiry could provide further theoretical insight into how untrained, naïve learners map the sounds of a second language onto the sounds of their native language while providing novel insight into whether these patterns can be altered as a result of short-term perceptual training.

Two groups of AE listeners completed a distributional learning task for the French /œ/-/o/ contrast as part of a larger study (Kabakoff *et al.*, 2020). An additional group of untrained listeners served as a control group. First, we hypothesized that both training groups would differ from the untrained group in their assimilation patterns for the novel /œ/ vowel and that learning would extend to the untrained, non-native /y/. This would be consistent with Levy (2009a), who found an effect of L2 experience on assimilation patterns. Second, we hypothesized that we would find evidence of greater learning for the bimodal group, due to previous research showing a bimodal advantage (Baese-Berk, 2010; Hayes, 2003; Maye and Gerken, 2000; Maye *et al.*, 2002). However, we also considered that both training conditions could result in similar perceptual assimilation patterns if accuracy feedback during training mitigates any differences between the two conditions, as found by Kabakoff *et al.* (2020) who demonstrated that group differences were only present in the initial stages of learning, but eliminated by the end of training.

## 2. Methods

### 2.1 Participants

The study included three groups of adult participants, ages 18–30: (1) trained on the unimodal distribution (n = 17, mean age 22.2), (2) trained on the bimodal distribution (n = 16, mean age 22.5), and (3) untrained control group (n = 16, mean age 18.9). All participants were native AE speakers with no history of speech or hearing disorders. All participants passed a hearing screening at 500, 1000, 2000, and 4000 Hz at 25 dB hearing level, and none had studied French or another language that included front-rounded vowels.

### 2.2 Materials

A 29-year-old male Parisian French speaker was recorded producing five repetitions of French vowels **/**œ/, /o/, /y/, /u/, /i/, and /ɛ/ in a /dVt/ context embedded in a phonotactically legal French nonword in a carrier phrase [“J'ai dit ___ à des amis” *I said ___ to some friends* (Levy, 2009a)]. The productions of /œ/ and /o/ that were most similar in duration and first formant frequency and were the farthest apart in terms of the second formant frequency were selected as the base vowels for synthesis of an 8-step training continuum. This /œ/-/o/ contrast was chosen due to the documented difficulty that AE listeners exhibit when trying to discriminate these speech sounds (Gottfried, 1984; Levy, 2009a,b; Levy and Strange, 2008). For more information on the synthesis of this continuum, see Kabakoff *et al.* (2020). For the perceptual assimilation task, the four French vowels /y/, /u/, /i/, and /ɛ/ that had durations closest to /œ/ and /o/ were also used. In addition to these six naturally produced vowels, the two resynthesized stimuli that were used as steps 1 and 8 on the acoustic continuum were included in the perceptual assimilation task.

### 2.3 Procedure

Participants in the unimodal and bimodal learning groups first completed two consecutive days of distributional training interspersed with identification and discrimination tasks that assessed learning of the /o/-/œ/ contrast. For the distributional training portion, participants listened to a block of 48 tokens from the eight-step synthetic /œ-o/ continuum four times. The number of times each stimulus was presented conformed to either a bimodal or unimodal distribution, as depicted in Fig. 1. Participants in the unimodal condition heard a majority of stimuli drawn from the center of the continuum, whereas participants in the bimodal condition heard a majority of stimuli drawn from the endpoints of the continuum. For each trial, listeners heard a stimulus and labeled it by selecting one of two shapes. Participants received accuracy feedback (correct/incorrect), such that steps 1–4 corresponded with one shape and steps 5–8 corresponded with a different shape. The mapping between steps and shapes was counterbalanced across participants. Each training block took approximately five minutes.

For participants assigned to the experimental conditions, the perceptual assimilation task was administered on the second day following all training blocks. Although we did not administer the perceptual assimilation task prior to training, which would be needed to look directly at perceptual changes following training, we did administer this same task to a group of untrained participants. The untrained, control participants received no training and completed the perceptual assimilation task in one visit. In the perceptual assimilation task, participants heard three instances of natural productions of /dœt/, /dot/, /dit/, /dɛt/, /dyt/, /dut/, and synthetic versions of /œ/ and /o/ (steps 1 and 8) in random order, resulting in 24 total trials. The rationale for including additional stimuli beyond those that were targeted in the training was twofold. First, we followed the precedent set by previous research which included additional vowels (Escudero and Boersma, 2002; Levy, 2009a). Second, it was important for there to be a range of vowels, including some that the participants would be confident in classifying (/i, ɛ/) and some that would be more challenging (/œ, o/ and /y, u/). As such, our primary prediction was that perceptual assimilation patterns following training would be different in the trained groups from in the control group for the trained contrast, but we were also interested in whether perceptual assimilation patterns following training would impact the analogous high vowel pair (/y, u/).

After each stimulus was presented, eight words appeared on a computer screen: “heed,” “hid,” “head,” “hud,” “hood,” “who would,” “heard,” and “hoed,” containing the AE vowels /i/, /ɪ/, /ɛ/, /ʌ/, /ʊ/, /u/, /ɝ/, and /o/. These target options were based on those found in Levy (2009b), as well as on pilot testing. Participants selected the word with the vowel that best matched the French stimulus by pressing keys 1–8 corresponding with the choices shown on the screen. Trials were separated by 1500 ms. This task took approximately five minutes to complete.

## 3. Results

Our analysis compared perceptual assimilation patterns between the bimodal, unimodal, and untrained groups. Specifically, this included analysis of how each group mapped each French vowel stimulus onto the set of AE vowels. Performance on the perceptual assimilation task was analyzed in R with the “rcompanion” package (Mangiafico, 2016) using Fisher's Exact Test, which allows examination of patterns of responses across groups when both the number of groups and the number of response options are greater than two. Fisher's test does not provide information about a particular pattern within a group (e.g., whether responses are varied or concentrated on a single response); rather, it provides information about whether the pattern of responses differs across groups. Because our overarching question was whether the three groups differed from each other in their distribution of responses for each vowel, 24 Fisher's tests were conducted (3 group comparisons × 8 vowels). The p-values were adjusted with the Benjamini Hochberg method.

The pairwise comparisons using Fisher's exact test are provided in Table 1. In response to our first question, a general effect of training was observed in that both trained groups performed differently from the untrained group for the natural /œ/ and /o/ vowels. Additionally, a significant difference was found between the bimodal and untrained groups for /ɛ/ and between the unimodal and untrained groups for the synthetic /o/ (step 8). In response to our second question, the two trained groups performed significantly differently from each other for the French /œ/ and /u/.

**Table 1.** Pairwise Fisher's exact tests among groups for the French vowels. p-values are corrected using the Benjamini Hochberg method; significant tests are those with p < 0.05 (boldface).

/i/	Bimodal – Unimodal	p = 0.370 387
	Bimodal – Untrained	p = 0.98 207
	Unimodal – Untrained	p = 0.553 832
/ɛ/	Bimodal – Unimodal	p = 0.661 68
	**Bimodal – Untrained**	**p = 0.004 071**
	Unimodal – Untrained	p = 0.070 597
/œ/	**Bimodal – Unimodal**	**p = 0.017 25**
	**Bimodal – Untrained**	**p = 0.000 07**
	**Unimodal – Untrained**	**p = 0.017 25**
/y/	Bimodal – Unimodal	p = 0.142 298
	Bimodal – Untrained	p = 0.349 101
	Unimodal – Untrained	p = 0.154 788
/u/	**Bimodal – Unimodal**	**p = 0.017 25**
	Bimodal – Untrained	p = 0.732 981
	Unimodal – Untrained	p = 0.142 298
/o/	Bimodal – Unimodal	p = 0.228 224
	**Bimodal – Untrained**	**p = 0.005 388**
	**Unimodal – Untrained**	**p = 0.004 071**
Synthetic /œ/ (Stim1)	Bimodal – Unimodal	p = 0.142 298
	Bimodal – Untrained	p = 0.240 225
	Unimodal – Untrained	p = 0.556 536
Synthetic /o/ (Stim8)	Bimodal – Unimodal	p = 0.693 545
	Bimodal – Untrained	p = 0.079 675
	**Unimodal – Untrained**	**p = 0.017 25**

Table 2 shows the percentage of listeners from each group who mapped a given French vowel onto an AE vowel. Closer inspection of the significantly different assimilation patterns revealed that the trained groups showed more heterogeneity in their selections for the French /œ/ (/i/, /ɪ/, /ɛ/, /ʌ/, /ʊ/, /u/, /ɝ/, /o/ for bimodal; /ɪ/, /ɛ/, /ʌ/, /ʊ/, /u/, /ɝ/, /o/ for unimodal) than the untrained group who only selected among /ʌ/, /ʊ/, /u/, /o/. For French /o/, the trained groups mapped this vowel onto AE /o/ the majority of the time (63% bimodal; 65% unimodal), whereas the untrained group had more varied responses with only 25% mapped to /o/ and 37.5% mapped to /ʊ/. A similar pattern was observed for synthetic /o/, where the trained groups selected /o/ on more than 60% of trials, whereas the untrained group selected /o/ on fewer than 40% of trials. This difference reached significance for only the unimodal group and approached significance for the bimodal group (corrected p = 0.0796).

**Table 2.** How French vowels (left column) were mapped onto American English vowels (top row) by AE listeners.

Stimulus	Group	/ i /	/ ɪ /	/ ɛ /	/ ʌ /	/ ʊ /	/ u /	/ ɝ /	/ o /
/ i /	Bimodal	70.8	27.1	4.2	2.1	—	2.1	—	—
	Unimodal	54.2	41.7	2.1	—	—	—	2.1	v
	Untrained	64.6	29.2	4.2	2.1	—	—	—	—
/ ɛ /	Bimodal	8.3	12.5	81.3	4.2	—	—	—	—
	Unimodal	4.2	6.3	87.5	2.1	—	—	—	—
	Untrained	—	—	100	—	—	—	—	—
/ œ /	Bimodal	6.3	10.4	10.4	22.9	33.3	2.1	16.7	4.2
	Unimodal	—	6.3	6.3	14.6	54.2	10.4	4.2	4.2
	Untrained	—	—	—	33.3	47.9	14.6	—	4.2
/ y /	Bimodal	6.3	8.3	—	29.2	8.3	52.1	2.1	—
	Unimodal	8.3	16.7	—	22.9	20.8	25	6.3	—
	Untrained	—	10.4	—	29.2	18.8	39.6	—	2.1
/ u /	Bimodal	—	—	—	12.5	39.6	52.1	2.0	—
	Unimodal	—	—	—	6.3	12.5	79.2	—	2.1
	Untrained	—	—	—	12.5	29.2	56.3	—	2.1
/ o /	Bimodal	—	—	—	12.5	12.5	12.5	6.3	62.5
	Unimodal	—	—	—	2.1	16.7	16.7	—	64.6
	Untrained	—	2.1	—	14.6	37.5	20.8	—	25
Synthetic /œ/ (Stim1)	Bimodal	2.1	4.2	2.1	35.4	33.3	10.4	12.5	6.3
	Unimodal	—	2.1	10.4	20.8	47.9	14.6	4.2	—
	Untrained	—	4.2	2.1	29.2	41.7	20.8	2.1	—
Synthetic /o/ (Stim8)	Bimodal	—	2.1	2.1	10.4	14.6	10.4	4.2	62.5
	Unimodal	—	2.1	—	4.2	12.5	16.7	—	64.6
	Untrained	—	—	—	8.3	41.7	10.4	2.1	37.5

Looking more closely at the significant differences between the bimodal and unimodal groups, we observed that more than half of the unimodal group (54%) labeled /œ/ as most similar to the AE /ʊ/, whereas for the bimodal group, the responses were distributed across /ʊ/ (33%), /ʌ/ (23%), and /ɝ/ (17%). Additionally, the majority of the unimodal group mapped French /u/ onto AE /u/ (79%), whereas the bimodal group mapped it onto AE /u/ (52%) or AE /ʊ/ (40%).

## 4. Discussion

The purpose of this study was to evaluate whether perceptual assimilation patterns of AE listeners would be impacted by perceptual training on the French /œ/-/o/ vowel contrast, and whether listeners trained on a bimodal versus unimodal distribution would differ from one another. Overall, both trained groups differed from the untrained group for the trained French vowels (/œ/ and /o/). Furthermore, the bimodal group and unimodal groups differed from each other in their perceptual assimilation patterns for /œ/ and /u/. Below we discuss the implications of these findings.

In response to our first question regarding general effects of training, our results suggest that perceptual training changed the way AE listeners assimilate French vowels onto their native vowel categories. General perceptual training effects (in which the bimodal and unimodal groups patterned differently from the control group) were seen for the French vowels /œ/ and /o/, consistent with our hypothesis that our training would specifically affect the trained vowels. Consistent with PAM (Best, 1995), it can be inferred that listeners in the bimodal and unimodal groups had acquired the /œ/-/o/ perceptual contrast. For the untrained listeners, the plurality of responses for both French /o/ and French /œ/ was AE /ʊ/ (47.9% and 37.9%, respectively), suggesting that they assimilated these two French vowels to the same English vowel, albeit /ʊ/ not /o/. This pattern of responses suggests that this group had difficulty discriminating between the two French vowels (Best, 1995). In contrast, both trained groups mapped French /o/ onto AE /o/ the majority of the time (>62%). Although a significant effect of training was found for both French /œ/ and French /o/, we were surprised to find that the training-induced mapping patterns for French /o/ were more robust than for French /œ/. That is, AE /ʊ/ was the modal response for all groups for the /œ/ stimuli, but the training groups showed dramatically higher mappings of French /o/ onto AE /o/ than the untrained group. This observation that groups showed selective differences in perceptual assimilation for one of the trained vowels (/o/) but not for the other (/œ/) strongly suggests that listeners must have learned (or at least started to learn) the difference between the non-native speech sounds.

The different patterns for the untrained listeners compared to the trained listeners is consistent with previous research showing an effect of language experience on perceptual assimilation patterns (Escudero and Boersma, 2002; Levy, 2009a). Interestingly, our result that the untrained group mapped both French /œ/ and French /o/ vowels onto the same AE vowel (/ʊ/) suggests that this would be a difficult sound contrast to learn, consistent with PAM (Best, 1995) and PAM-L2 (Best and Tyler, 2007). However, we cannot rule out the possibility that the untrained control group readily discriminated between these two French vowels, perceiving one as a better exemplar of /ʊ/ than the other, but still mapped them onto the same AE vowel. Future research in this domain should include a category goodness task to explore this possibility.

One surprising finding was that even though all three groups had their largest percentage of French /œ/ mapped onto English /ʊ/, the responses were actually *more* heterogeneous for the two trained groups, with seven and eight vowels selected for these two groups, in comparison to the untrained group with only four vowels selected. The previous studies that examined patterns found a reduction in mapping, where listeners with more exposure narrow their responses (Escudero and Boersma, 2002; Levy, 2009a).

With respect to our second question regarding differences between the trained groups, we found some evidence for an effect of training condition. For French /œ/, the group whose perceptual assimilation pattern differed more from the untrained group would be interpreted as having learned the non-native contrast best. Our original hypothesis was that the bimodal group would show more two-category assimilation patterns because the bimodal group could be interpreted as representing listeners with more language experience. If new speech sound categories are formed based on experience (Best and Tyler, 2007; Flege, 1995), then a finding consistent with our hypothesis would be that the bimodal group would show more homogeneous responses when mapping French /œ/ onto AE vowels. However, the present results indicate that the unimodal group showed more response homogeneity by selecting only three vowels (/ʌ/, /ʊ/, /u/) for more than 80% of trials (each with at least 10% of responses), and that this pattern was akin to that found for the untrained group, who selected the same three vowels for more than 90% of trials. In contrast, the bimodal group showed a more heterogeneous response pattern by selecting five vowels with at least 10% of responses (/ɪ/, /ɛ/, /ʌ/, /ʊ/, /ɝ/). This finding in which the unimodal group showed more homogeneity in responses should indicate that the unimodal group learned more than the bimodal group. However, we also acknowledge that increased response heterogeneity could indicate that those in the bimodal group had learned the non-native contrast so well that they knew that /ʊ/ was not an ideal selection, so they became more likely to select other vowels that also were not ideal matches with /œ/. As the design of our study was not equipped to tease apart the differential perceptual assimilation effects of the bimodal versus unimodal groups, we leave this investigation to future research. Another limitation to the interpretation of this result is that the degree of language exposure was limited to short laboratory-based training sessions, which is not equivalent to the real-life language experience that has been examined in previous studies (Escudero and Boersma, 2002; Levy, 2009a). Future research should include longer periods of perceptual training in order to better approximate the effects of authentic language experience.

Although our two questions were about the perceptual assimilation patterns of our three groups, we observed some unexpected effects for untrained vowels. First, perceptual assimilation of the French /ɛ/ was found to differ between the bimodal and untrained groups. Upon inspection of this pattern, it was observed that the untrained group showed more restricted responses, with 100% of responses for AE /ɛ/, whereas both the bimodal and unimodal groups responded with less than 90% for this same AE vowel, while also selecting other front vowels. Second, the bimodal and unimodal groups were found to differ in their response patterns for the French /u/. The unimodal group showed more homogeneous responses, selecting AE /u/ on nearly 80% of trials, whereas the bimodal group showed more response heterogeneity with relatively equal selection of AE /u/ or /ʊ/. Given that neither of these French vowels were trained, both of these significant effects were unexpected and did not conform to our theoretical predictions. Generally, we interpret these effects as byproducts of the redistribution of responses that may have occurred as a result of the training. That is, the response variability that occurred as a result of the training on the /œ/-/o/ contrast led to a redistribution of how other sounds were mapped onto the same selection of sounds. We leave it to future research to discern how the entire vowel system may be remapped following training on a non-native contrast.

Taken together, our findings suggest that perceptual training can change how listeners map the speech sounds of a non-native language onto the speech sound categories of their native language. As indicated by the general effects of perceptual training on perceptual assimilation patterns relative to the untrained group, training using both the unimodal and bimodal distributions resulted in increases in two-category perceptual assimilation patterns. However, our results do not clearly suggest that exposure to a bimodal distribution leads to more learning than exposure to a unimodal condition. In educational settings, a distributional training paradigm could be implemented in a variety of ways including incorporation into a training protocol to help those learning a second language to become more perceptually attuned to the speech sound contrasts of their target language. Such a paradigm may even be useful for individuals with perceptual deficits associated with speech sound disorders.
